# Explainable Machine Learning Using Sensor-Derived Biomechanical Features to Classify Elevated VALR-Related Loading Across Midsole Hardness Conditions in School-Aged Boys

**DOI:** 10.3390/s26123942

**Published:** 2026-06-21

**Authors:** Yiyao Chen, Zixiang Gao, Fengping Li, Dongxu Wang, Jianqi Pan, Yucheng Wang, Diwei Chen, Zhanyi Zhou, Lidong Gao, Kuiyu Chen, Zhaolong Ye, Yaodong Gu

**Affiliations:** 1 Faculty of Sports Science, Ningbo University, Ningbo 315211, China; 2511040003@nbu.edu.cn (Y.C.); 2211040065@nbu.edu.cn (F.L.); 2311040022@nbu.edu.cn (D.W.); 2411040017@nbu.edu.cn (J.P.); 2411040024@nbu.edu.cn (Y.W.); 2311040040@nbu.edu.cn (D.C.); 2511040036@nbu.edu.cn (K.C.); 2511040108@nbu.edu.cn (Z.Y.); 2 Faculty of Kinesiology, University of Calgary, Calgary, AB T2N 1N4, Canada; 3 Department of Physical Education and Sport, Faculty of Sport Sciences, University of Granada, 18011 Granada, Spain; zhanyizhou@correo.ugr.es; 4 Department of Material Science and Technology, Audi Hungaria Faculty of Automotive Engineering, Széchenyi István University, 9026 Győr, Hungary; gao.lidong@sze.hu; 5 Department of Kinesiology, Hungarian University of Sports Science, 1123 Budapest, Hungary

**Keywords:** school-aged boys, midsole hardness, forefoot strike, elevated VALR-related loading, XGBoost, SHAP

## Abstract

**Highlights:**

**What are the main findings?**

- Vertical average loading rate (VALR) showed an overall increasing trend with increasing midsole hardness, but no statistically supported change point was identified. Elevated-loading labels were therefore defined using trial-level VALR values rather than a fixed midsole hardness threshold.
- Under participant-level GroupKFold validation, XGBoost showed moderate classification performance for elevated VALR-related loading, with an accuracy of 75.93%, F1-value of 0.768, and pooled out-of-fold AUC of 0.738. Exploratory SHAP analysis indicated that distal and non-sagittal kinematic features contributed most to model classification, followed by sEMG features and proximal sagittal kinematic features.

**What are the implications of the main findings?**

- The findings do not support defining 47 Shore C as a critical midsole hardness threshold, suggesting that midsole hardness evaluation in children should consider the overall VALR response rather than a fixed cut-off point.
- The explainable machine-learning framework provides an exploratory model-based approach for identifying multi-feature biomechanical patterns related to elevated VALR-related loading, but the findings should be validated in larger and more diverse pediatric samples.

**Abstract:**

(1) Background: Changes in midsole hardness may affect lower-limb impact loading during forefoot strike (FFS) running in children, yet the biomechanical basis for discriminating elevated VALR-related loading remains unclear. (2) Methods: Fourteen school-aged boys performed FFS running tests in experimental shoes with four midsole hardness levels (37, 42, 47, and 52 Shore C). Lower-limb kinematics and surface electromyography (sEMG) data were collected during the dominant leg stance phase. After preprocessing, VALR was calculated from 336 valid trials, and 28 stance-phase biomechanical features were extracted, yielding a final machine-learning dataset of 324 trials after excluding incomplete feature data. VALR was used to compare loading changes and define trial-level elevated-loading labels based on the median VALR value. Classification models were evaluated under participant-level GroupKFold validation, and XGBoost was retained for exploratory SHAP analysis. (3) Results: VALR showed an upward trend with increasing hardness, but no statistically supported change point was identified. XGBoost achieved an accuracy of 75.93%, precision of 74.14%, recall of 79.63%, F1-value of 0.768, and pooled out-of-fold AUC of 0.738. SHAP analysis indicated that distal and non-sagittal kinematic features contributed most to model classification. (4) Conclusions: Elevated VALR-related loading during children’s FFS running may be characterized by a multi-feature model-based pattern rather than a fixed midsole hardness threshold.

## 1. Introduction

School-aged children are in a period of rapid growth and development, during which the bones, joints, ligaments, and muscles of the lower limbs are not yet fully developed. As a result, their functional and morphological characteristics differ substantially from those of adults [1]. Previous studies show that running in children is not simply a scaled-down version of adult running. Although walking becomes relatively mature after the age of five, the running pattern of children remains markedly immature throughout the early school-age period, and the typical adult spring–mass mechanism usually does not begin to emerge until around the age of nine [2]. Compared with adults, children generally exhibit a higher vertical average loading rate (VALR) and greater joint moments. When coping with impact, they tend to maintain movement rhythm rather than adopt an active cushioning strategy by prolonging the stance phase [3]. In this context, shoes, as the primary interface between children and the ground, affect foot health and gait performance, and different sole hardness levels do not play the same role in foot and gait development [4]. The sole, including the midsole and outsole, is functionally central to footwear design. To some extent, an appropriate midsole hardness provides effective cushioning, reduces the impact generated at foot contact during movement, and alleviates local discomfort [5]. A softer midsole is considered a key strategy for reducing loading rate and improving cushioning, which may help prevent sports injuries [6,7]. Although forefoot strike (FFS) is regarded as an effective strategy for preventing running-related injury because it reduces the vertical loading rate at initial contact [8,9,10], evidence shows that injury distribution in adolescent FFS runners presents a clear proximal shift, with the proportions of hip and knee injuries being significantly higher than those in other foot strike patterns [11]. This finding suggests that, while FFS provides distal buffering, it may also induce a redistribution of kinetic chain loading toward proximal segments, which may be associated with a specific risk mechanism in developing children. Previous studies show that, compared with barefoot running, shoe-wearing alters gait patterns in children by restricting joint range of motion, and changes in sole hardness affect plantar pressure distribution and internal neuromuscular control strategies [4,12]. Surface electromyography (sEMG), as a core tool for quantifying muscle activity, directly reflects internal work production and active compensation when the body responds to external impact loading [13]. Earlier studies indicate that changes in running shoe midsole hardness significantly affect the external loading characteristics of the lower limbs [14,15], while sEMG variables effectively quantify muscle activation level and neuromuscular control strategy during movement [16,17]. However, current studies on running in developing children under different midsole hardness conditions still focus mainly on spatiotemporal parameters, local loading, or isolated biomechanical variables [11]. A systematic understanding of the coordinated effects of multidimensional features, including kinematics, kinetics, and electromyography, and of their relationship with changes in elevated VALR-related loading remains limited [18,19]. Therefore, it remains unclear whether elevated VALR-related loading in children under different midsole hardness conditions is better characterized by a single hardness-related indicator or by a coordinated multi-feature biomechanical pattern. From the perspective of load regulation within the lower-limb kinetic chain, impact absorption during the stance phase of running mainly depends on sagittal-plane flexion and extension coordination of proximal joints such as the hip and knee, because this process determines how the body buffers ground reaction force and absorbs energy [20]. When this dominant cushioning strategy is constrained, the body often maintains gait stability and redistributes mechanical load through non-sagittal control of the hip and adjustments in distal structures, including the knee, ankle, and forefoot [20]. Therefore, biomechanical responses of children during running under different midsole hardness conditions may not only be reflected in changes in proximal sagittal-plane movement strategies but may also involve multiplanar and multijoint compensatory adjustments [21]. In FFS running, this process of load redistribution may be even more pronounced. Given the distinct clinical injury pattern associated with FFS in adolescents [11], ignoring how this specific running pattern is modulated by hardness may lead to misjudgment of the evolving trend of key loading indicators such as VALR, which in turn may compromise accurate identification of changes in VALR-related loading during children’s running. As a core biomechanical parameter for evaluating impact loading, abnormal variation in VALR often indicates the limit of midsole cushioning performance and potential exercise-related risk [22]. Therefore, this study focuses on the FFS running pattern in children and uses VALR as the key indicator to evaluate changes in potential loading-related concerns under shoes with different midsole hardness levels.

In running biomechanics, lower-limb kinematic, kinetic, and muscle activation data collected at foot contact are highly dimensional and exhibit complex nonlinear patterns [23]. Because children in the developmental stage show substantial inter-individual differences and multisegment compensatory responses when coping with impacts under different hardness conditions, traditional linear statistical methods often struggle to detect subtle shifts in movement patterns from a large number of variables and may even obscure key risk-related features. Machine learning methods can use large-scale movement data from traditional laboratories, rehabilitation clinics, and wearable sensors to characterize and predict patterns of healthy and pathological movement [23]. In biomechanics research, commonly used models for classification include logistic regression (LR), K-nearest neighbor (KNN), random forest (RF), multilayer perceptron (MLP), and gradient-boosted tree models [24]. These models represent different methodological families, including linear classification, instance-based learning, neural network modeling, and ensemble tree learning, and they characterize the relationship between multidimensional biomechanical features and elevated VALR-related loading from different perspectives. Among them, XGBoost improves predictive performance by combining weak learners and minimizing model residuals [25]. Compared with some conventional machine learning methods, XGBoost relies on a regularized additive tree ensemble and can be useful for modeling nonlinear relationships, capturing feature interactions, and controlling model complexity. It is therefore widely used in classification tasks on structured tabular data [26]. In structured tabular classification tasks, XGBoost has shown competitive performance and robustness in many applications while requiring relatively limited hyperparameter tuning. Previous research uses XGBoost to predict fall risk in older adults and obtains good predictive performance using gait variables alone while also identifying key gait features [27], which demonstrates its suitability and interpretability in multivariable biomechanical settings. However, such ensemble learning models generally have a black-box nature, because gains in predictive accuracy are often accompanied by reduced transparency in the decision process [28], which raises concerns about reliability in some movement-related applications. To address this limitation, this study incorporates the explainable artificial intelligence method Shapley Additive Explanations (SHAP). By combining the high predictive performance of XGBoost with the game-theoretic explanation mechanism of SHAP, the actual contribution of each key variable to the model’s classification outcome can be directly revealed, thereby improving the interpretability and auditability of complex algorithms [29].

Against this background, the present study combines high-precision kinematic measurement and surface electromyography to systematically examine lower-limb biomechanical response characteristics during the stance phase of the dominant leg in school-aged boys performing forefoot strike running under different midsole hardness conditions. VALR is used as the key indicator to characterize lower-limb impact loading and changes in VALR-related loading. On this basis, an XGBoost binary classification model is employed to identify biomechanical response patterns under different loading states, and SHAP is further used to interpret the key discriminative features. This study aims to examine VALR-related loading responses and associated biomechanical feature patterns during the running stance phase across midsole hardness conditions in children and to provide preliminary evidence for evaluating midsole hardness and multidimensional biomechanical responses in children’s athletic footwear.

## 2. Materials and Methods

### 2.1. Participants

Based on previous research [30], the effect size is set at f = 0.45, with α = 0.05 and power = 0.85. A priori power analysis using G*Power 3.1.9.7 indicates that the minimum required sample size is 12. Accordingly, this study recruits 14 school-aged boys aged 7 to 12 years (age: 10.31 ± 2.62 years; height: 145.39 ± 19.10 cm; body mass: 39.99 ± 16.01 kg; BMI: 18.16 ± 3.27 kg/m^2^). Age was calculated from the recorded birth date to the testing date in 2025, and BMI was calculated as body mass divided by height squared. Only boys were included to reduce the potential influence of sex-related differences in growth, maturation, lower-limb biomechanics, and neuromuscular control on the small-sample analysis [31]. This design allowed the present study to examine the effect of midsole hardness under relatively homogeneous participant characteristics. Future studies should include girls and further examine possible sex-related differences. The inclusion criteria are as follows. The children show normal development, have no foot deformity, no medical condition or disease history that may affect gait, no history of lower-limb injury, and do not engage in strenuous exercise within 48 h before testing [5,19,32]. They present a normal gait pattern, with no obvious abnormality in walking or running. In this study, a normal gait pattern was defined as the absence of visually observable abnormalities during walking and running, including obvious limping, asymmetric step pattern, excessive foot inversion or eversion, toe-in or toe-out gait, or pain-related movement compensation. This was screened by the experimenters during the familiarization trials before formal data collection [33]. Their dominant leg is the right leg. Their foot length matches the experimental shoes. All participants regularly attended school physical education classes, and none was reported by parents or guardians to have received systematic professional running training. Written informed consent is obtained from a parent or legal guardian. The study protocol is approved by the Ethics Committee of Ningbo University (Approval No. TY2025065).

### 2.2. Experimental Shoes

**Figure 1:**
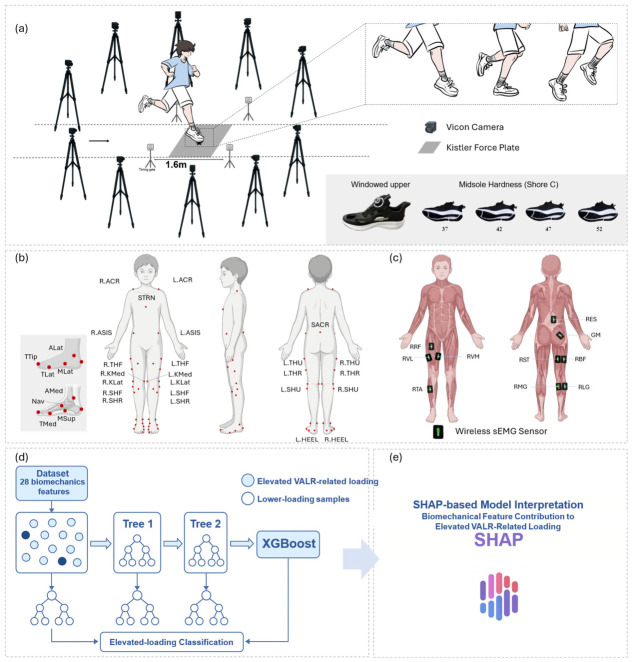
Schematic illustration of motion capture, biomechanical feature extraction, XGBoost classification, and SHAP-based model interpretation. (**a**) Experimental setup for motion data collection and the test shoes; (**b**) placement of reflective markers; (**c**) placement of sEMG sensors; (**d**) musculoskeletal model and extraction of biomechanical parameters; (**e**) workflow of XGBoost-based elevated-loading classification and SHAP interpretation analysis using biomechanical features.

This study uses children’s running shoes with identical appearance but different midsole hardness levels of 37, 42, 47, and 52 Shore C. All experimental shoes were the same model of children’s running shoes provided by ANTA Sports Products Limited, Jinjiang, China. The shoes had identical upper structure, outsole pattern, shoe last, and external appearance, and only the midsole hardness differed among the four conditions. This design was used to reduce the influence of shoe geometry and material differences other than midsole hardness. Midsole hardness is measured on the material surface using a Shore C durometer, and the shoes are grouped according to the measured values [5]. The appropriate shoe size is selected according to foot length. Circular openings are made in the upper part of each shoe to allow reflective markers to be placed directly on the skin surface [34] (Figure 1a).

### 2.3. Experimental Procedure

A Vicon three-dimensional motion capture system with 10 cameras (Oxford Metrics Ltd., Oxford, UK) and a Kistler force platform (Kistler Instruments AG, Winterthur, Switzerland) are used in this study. Before testing each participant, the Vicon system was calibrated using the manufacturer-recommended calibration procedure to ensure marker tracking accuracy. Data collection was started only after the calibration quality met the system requirements. Based on a marker model with 40 reflective markers (Figure 1b), kinematic and kinetic data during the stance phase of the dominant leg are synchronously collected while the participants run in the experimental shoes with different midsole hardness levels. The sampling frequencies are 250 Hz for motion capture and 1000 Hz for force data. The dominant leg is defined as the leg used in the ball-kicking task [35].

After each participant understands the test movements and becomes familiar with the experimental setting, the formal experiment begins. A randomized double-blind design is adopted to control for order effects, and each participant wears the experimental shoes with midsole hardness levels of 37, 42, 47, and 52 Shore C in a random order. Because the four shoe conditions had identical external appearance, participants were not informed of the specific midsole hardness condition during testing. The shoe conditions were coded before data collection, and the experimenters responsible for data processing and statistical analysis used only the coded condition labels. Thus, participants and data analysts were blinded to the hardness condition, whereas the staff member responsible for shoe preparation was not involved in data processing or statistical analysis. After the shoes are fitted, the participant stands on the force platform with the feet shoulder-width apart for static calibration. Formal testing then starts. After the start command is given, the child begins from the starting line and runs through the 1.6 m photoelectric timing gate at a relaxed jogging pace. Running speed was monitored using a Brower TCi photoelectric timing system (Brower Timing Systems, Draper, UT, USA) placed along the runway. The system consisted of paired photoelectric transmitter and receiver units, and the running time was recorded when the participant interrupted the light beam. The same timing system, gate distance, and placement were used for all participants and all shoe conditions to ensure consistent speed control. To ensure a relatively consistent running speed across trials, the time required to pass through the 1.6 m timing gate was controlled between 0.7 and 0.8 s, corresponding to an approximate running speed range of 2.0–2.3 m/s. Each condition is repeated eight times. At least six valid trials are obtained for each participant under each condition. To reduce fatigue, participants rested for at least 30 s between trials and for at least 3 min between different shoe conditions. Additional rest was provided when a participant reported fatigue or when the experimenter observed decreased running quality, such as obvious slowing, unstable foot placement, or failure to maintain the required running path. The formal test was continued only after the participant reported readiness to proceed. A trial is considered valid when the dominant foot lands completely within the effective area of the force platform, the participant passes through the timing gate along the predefined straight path without deviating from the path or slowing down to turn, and no reflective marker is detached during data collection. After testing under one hardness condition is completed, the shoes are changed, and the procedure continues until all shoe conditions have been tested.

### 2.4. Data Preprocessing and Feature Extraction

The experimental data are processed in Vicon Nexus 2.6.1 (Vicon Motion Systems, Oxford, UK) for marker labeling, trajectory identification, and gap filling. The data are exported in c3d format and then imported into MATLAB R2023a (MathWorks, Natick, MA, USA) and OpenSim 4.4 (Stanford University, Stanford, CA, USA) for further processing. To remove high-frequency noise, a Butterworth low-pass filter is applied to the synchronized multimodal signals. The cut-off frequency is set at 6 Hz for kinematic data. For the ground reaction force data collected synchronously from the Kistler force platform, the cut-off frequency is set at 50 Hz, after which the force data are aligned with the kinematic data [36]. All landing patterns in this experiment are FFS. Initial contact is identified from the vertical ground reaction force (vGRF), with the threshold set at 20 N [37]. The data for each stance phase are time-normalized to 101 frames.

Surface electromyography signals were collected using a Delsys wireless sEMG system (Delsys Inc., Natick, MA, USA) at a sampling frequency of 2000 Hz. Electrode placement strictly followed the recommendations of the SENIAM project. Before electrode placement, hair over the target area was removed when necessary, and the skin was cleaned with alcohol to reduce surface impedance. The electrodes were placed parallel to the direction of the muscle fibers over the most prominent part of the muscle belly [38]. To reduce electrode displacement and motion artifacts caused by landing impact, all sensors were fixed using dedicated double-sided EMG adhesive and a self-adhesive elastic bandage [38]. The synchronized surface electromyography (sEMG) signals are preprocessed in Python 3.10 (Python Software Foundation, Wilmington, DE, USA). After baseline correction, the raw signals are denoised with a fourth-order Butterworth band-pass filter at 20–450 Hz and then full-wave rectified. A fourth-order Butterworth low-pass filter at 15 Hz is subsequently used to extract the linear envelope, and the root mean square (RMS) values during the stance phase of the dominant leg are calculated. Because school-aged children often cannot perform maximal voluntary isometric contraction (MVC) tests consistently and previous studies show that maximal isometric contraction tests have relatively large between-subject variability and usually lower reproducibility than submaximal or dynamic tests [16,39], this study uses reference condition normalization. Specifically, the mean RMS value of each muscle for each participant under the softest midsole condition, 37 Shore C, is used as the baseline, and the RMS values under the other hardness conditions are divided by this baseline to obtain relative muscle activation levels [40]. This procedure reduces feature differences caused by variation in absolute muscle activity across muscles [41].

Based on the above procedures, a total of 28 core biomechanical features are extracted from the stance phase of the dominant leg and used as inputs to the subsequent XGBoost binary classification model. To systematically characterize biomechanical response patterns under different midsole hardness conditions, these features are grouped into three dimensions. The first dimension is EMG features, which reflect neuromuscular activation levels and include the RMS values of 10 muscles: semitendinosus (ST), medial gastrocnemius (MG), gluteus maximus (GMAX), erector spinae (ES), tibialis anterior (TA), biceps femoris (BF), vastus lateralis (VL), vastus medialis (VM), rectus femoris (RF), and lateral gastrocnemius (LG). The second dimension is proximal sagittal kinematics, which characterizes the basic gait trajectory of the lower limbs and includes the mean angles and ranges of motion (ROM) of the hip and knee joints in the sagittal plane [20]. The third dimension is distal and non-sagittal kinematics, which reflects multiplanar joint compensation and distal loading, and includes multidimensional spatial parameters such as knee valgus, metatarsophalangeal (MTP) joint motion, hip rotation and adduction, and subtalar joint motion [21]. To provide a clearer and more reproducible description of the extracted biomechanical variables, all 28 input features used for machine-learning classification are listed in Appendix A, Table A1.

### 2.5. VALR-Based Definition of Elevated-Loading Labels

This study uses VALR as the core mechanical indicator for defining elevated impact-loading labels associated with midsole hardness. VALR reflects the impact loading rate experienced by the lower limbs at the instant of foot contact and is widely used to assess mechanical loading characteristics during running [7,42]. VALR is defined as the average slope of the vertical ground reaction force (vGRF) between 20% and 80% of its first impact peak [7,22,42] and is calculated as follows:

(1) $$VALR=\frac{GRF_{80\%}-GRF_{20\%}}{t_{80\%}-t_{20\%}}$$

where $GRF_{80\%}$ and $GRF_{20\%}$ denote the values of the vertical ground reaction force at 80% and 20% of the impact peak, respectively, expressed in body weight (BW), and $t_{80\%}$ and $t_{20\%}$ denote the corresponding time points in seconds. All kinetic data are normalized to body weight. Given that no statistically supported change point was identified across midsole hardness conditions, the classification labels were defined using trial-level VALR values. Specifically, the median VALR value across all valid trials was used as the cut-off point for defining high-VALR loading. The median VALR cut-off value was 21.80 BW·s^−1^. Based on this cut-off, the final machine-learning dataset included 324 trials, with 162 elevated-loading samples and 162 lower-loading samples. Trials with VALR values greater than or equal to the median were labeled as elevated-loading samples (Class 1), whereas trials with VALR values below the median were labeled as lower-loading samples (Class 0). These labels served as the outcome variable for the subsequent machine-learning classification.

### 2.6. XGBoost Model Construction and Participant-Level Validation

To further capture the complex nonlinear relationship between multidimensional biomechanical indicators and elevated impact loading, this study employs XGBoost as the primary classification model [23]. To compare the suitability of different machine learning methods for identifying high-VALR loading during the stance phase of FFS running in school-aged boys under different midsole hardness conditions, and to evaluate the classification performance of XGBoost, logistic regression (LR), support vector machine (SVM), k-nearest neighbors (KNN), multilayer perceptron (MLP), and random forest (RF) are selected as baseline models [43,44]. To rigorously control the risk of overfitting, which is particularly pronounced for tree-based models in small-sample settings, this study adopts a participant-level GroupKFold cross-validation framework. All trials from the same participant are kept within the same fold and assigned exclusively to either the training set or the test set, thereby reducing information overlap among trials from the same child. In each iteration, model training and performance evaluation are strictly separated. Missing value imputation and feature standardization are fitted only on the training folds and then applied to the corresponding test fold, which reduces the risk of information leakage from the test set into the training process [45,46]. In addition, a nested GroupKFold cross-validation procedure is conducted within the training set for hyperparameter tuning, and grid search is used to determine the optimal hyperparameter combination [45,47]. The objective function of XGBoost consists of a loss term and a regularization term:

(2) $$L=\sum_{i=1}^{n}l\left(y_{i},{\hat{y}}_{i}\right)+\sum_{k=1}^{t}\Omega \left(f_{k}\right)$$

where $l\left(y_{i},{\hat{y}}_{i}\right)$ denotes the loss function for a single sample, and Ω(f _k_) denotes the structural regularization term of the *k* th regression tree, which controls model complexity and suppresses overfitting. During XGBoost training, the loss function is approximated by a second-order Taylor expansion, which transforms the original optimization problem into a quadratic form. This improves learning efficiency and enhances generalization ability. In the binary classification task, the model output score ${\hat{y}}_{i}$ is mapped to a posterior probability through the sigmoid function:

(3) $$P\left(y=1|x\right)=\frac{1}{1+exp\left(-\hat{y}\right)}$$

Because the positive class is defined according to high-VALR loading, a training-fold probability orientation check is performed to ensure that the predicted probability corresponds to the predefined elevated-loading label. The probability orientation is determined only within the training data and then applied to the held-out participant-level test fold. To verify the discriminative validity and robustness of the model, its performance is evaluated using Accuracy, Precision, Recall, F1 score, and the area under the receiver operating characteristic curve (AUC), and pooled out-of-fold AUC [48,49]. The definitions of these evaluation metrics are as follows:

(4) $$Accuracy=\frac{TP+TN}{TP+FP+TN+FN}$$

(5) $$Recall=\frac{TP}{TP+FN}$$

(6) $$F1=\frac{2\cdot Precision\cdot Recall}{Precision+Recall}$$

(7) $$Precision=\frac{TP}{TP+FP}$$

where TP (true positive) and TN (true negative) denote the numbers of elevated-loading and lower-loading samples correctly identified by the model, respectively, while FP (false positive) and FN (false negative) denote the numbers of lower-loading and elevated-loading samples incorrectly classified by the model, respectively [50].

### 2.7. SHAP Interpretability Analysis

To address the black-box nature of XGBoost [28], this study applies SHAP to decompose the model prediction into additive contributions. Based on cooperative game theory, the SHAP algorithm treats the model output as the total gain generated through the joint effect of multiple features and regards each input feature as a stakeholder [29,51]. By computing the marginal contribution of each feature across all possible feature combinations, SHAP provides a principled and fair allocation of credit, which enables an intuitive and objective quantification of the model-based contribution of each key biomechanical variable to the model output [24,51]. The mathematical expression is given as follows [52]:

(8) $$f\left(x\right)=\phi_{0}+\sum_{i=1}^{M}\phi_{i}x_{i}$$

where $\phi_{0}$ is the baseline prediction, and $\phi_{i}$ denotes the SHAP contribution of the i th feature. In the folds where probability orientation calibration was applied, SHAP values were aligned with the calibrated high-VALR loading probability. Therefore, SHAP values were interpreted as model-based feature contributions rather than causal biomechanical effects.

## 3. Results

### 3.1. VALR Response Across Midsole Hardness Conditions

Table 1 presents the VALR results during forefoot strike running under the four midsole hardness conditions. The mean VALR was 19.07 ± 4.56 $BW\cdot s^{-1}$ under the 37 Shore C condition and 20.13 ± 5.31 $BW\cdot s^{-1}$ under the 42 Shore C condition. Under the 47 Shore C condition, the mean VALR increased to 23.83 ± 10.50 $BW\cdot s^{-1}$, and under the 52 Shore C condition, the mean VALR was 24.02 ± 10.16 $BW\cdot s^{-1}$.

The largest descriptive increase in mean VALR was observed between 42 and 47 Shore C. However, adjacent pairwise comparisons showed no significant differences after Holm correction. As shown in Table 2, the candidate change-point analysis further indicated that the linear model without a change point had the lowest AIC compared with the piecewise models with candidate knots at 42 and 47 Shore C. Therefore, no statistically supported inflection point was identified, and the VALR response was interpreted as an overall increasing pattern across midsole hardness conditions.

### 3.2. Comparative Analysis of Model Performance Under Participant-Level Validation

**Figure 2:**
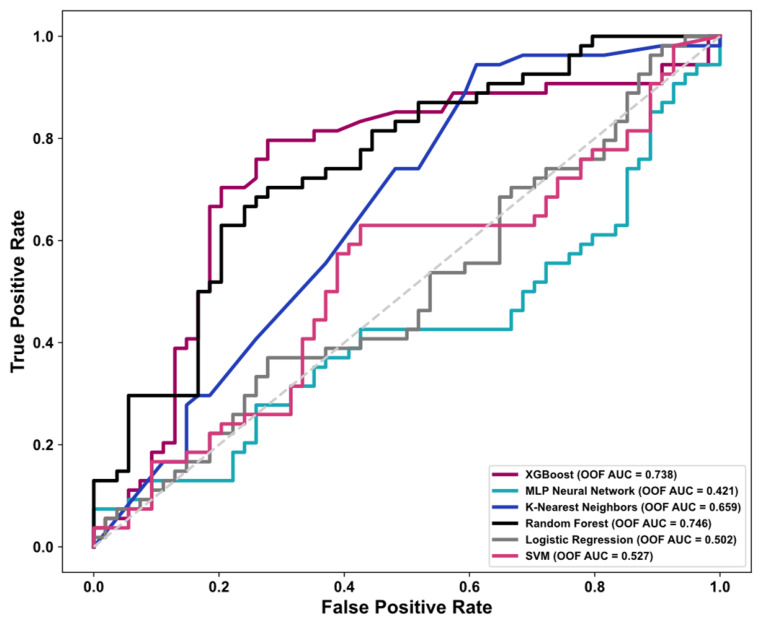
Comparison of ROC curves for different classification models under participant-level GroupKFold validation. The grey dashed diagonal line represents the no-discrimination reference line (AUC = 0.5). AUC values were calculated using pooled out-of-fold predictions.

The machine-learning classification analysis was conducted using the final dataset of 324 trials, including 162 elevated-loading samples and 162 lower-loading samples. Table 3 reports the elevated-loading classification performance of all models under participant-level GroupKFold validation, and Figure 2 presents the receiver operating characteristic (ROC) curves. The results show that XGBoost achieved the highest accuracy, precision, and F1-value among the tested models, whereas KNN showed the highest recall and Random Forest showed the highest pooled OOF AUC. The pooled OOF AUC of XGBoost reached 0.738, which is slightly lower than that of RF (0.746) but higher than that of KNN (0.659), SVM (0.527), LR (0.502), and MLP (0.421). In terms of accuracy, XGBoost attained 75.93%, which is also higher than that of the other comparison models. For the identification of elevated-loading trials, the precision, recall, and F1-value of XGBoost were 74.14%, 79.63%, and 0.768, respectively. Although its recall was lower than that of KNN, XGBoost showed the highest F1-value, indicating a better balance between precision and recall. From the distribution of the ROC curves, the XGBoost and RF curves lie closer to the upper-left corner overall than the other comparison models. Considering the overall classification metrics and their compatibility with SHAP-based interpretation, XGBoost was retained for the subsequent exploratory feature contribution analysis.

### 3.3. ROC Curve and Confusion Matrix of the XGBoost Model Under Participant-Level Validation

**Figure 3:**
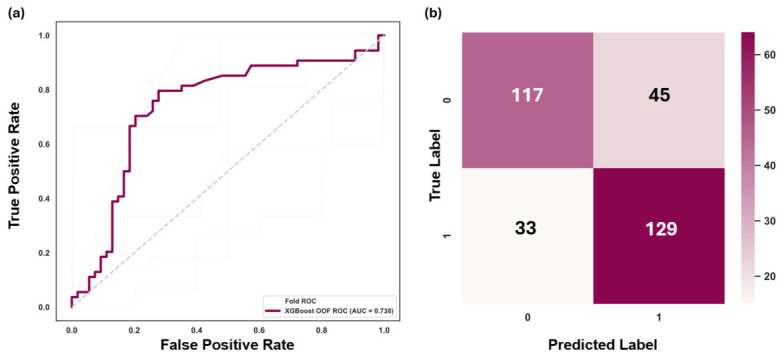
ROC curve and confusion matrix of the XGBoost model under participant-level GroupKFold validation. (**a**) ROC curve; (**b**) confusion matrix. Values were based on pooled out-of-fold predictions.

Figure 3 presents the ROC curve and confusion matrix of the XGBoost model under participant-level GroupKFold validation. The pooled out-of-fold ROC curve showed an AUC of 0.738. Fold-level ROC curves are displayed as faint background curves. The confusion matrix based on pooled out-of-fold predictions showed 117 true negatives, 45 false positives, 33 false negatives, and 129 true positives. The corresponding accuracy, precision, recall, and F1-value were 75.93%, 74.14%, 79.63%, and 0.768, respectively.

### 3.4. Exploratory SHAP-Based Feature Contribution Analysis

**Figure 4:**
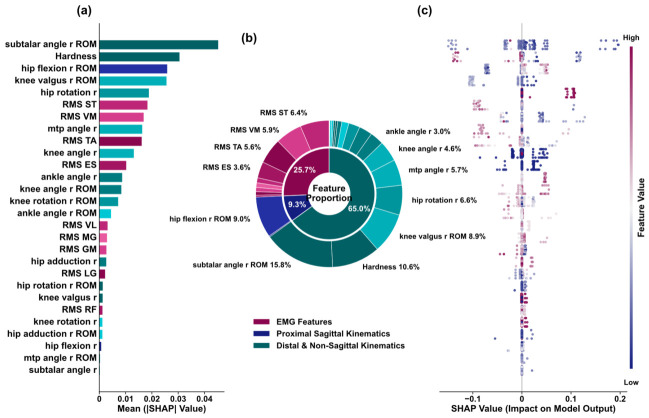
Visualization of exploratory SHAP feature contributions for the XGBoost model. (**a**) Biomechanical features ranked by mean absolute SHAP values. (**b**) Donut chart showing the proportional contribution of feature groups based on mean absolute SHAP values. (**c**) SHAP summary plot, where each point represents one sample and color indicates the feature value, with magenta denoting high values and blue denoting low values. Positive SHAP values indicate contributions toward classification as elevated VALR-related loading. Abbreviations: RMS, root mean square; ROM, range of motion; r, right; MTP, metatarsophalangeal; GM, gluteus maximus; TA, tibialis anterior; ST, semitendinosus; MG, medial gastrocnemius; ES, erector spinae; LG, lateral gastrocnemius; RF, rectus femoris; VM, vastus medialis.

Figure 4 presents the exploratory SHAP feature contribution results of the XGBoost model. The mean absolute SHAP values showed that distal and non-sagittal kinematic features accounted for the largest proportion of the model feature contribution, contributing 65.0% of the total mean absolute SHAP value. EMG features accounted for 25.7%, whereas proximal sagittal kinematic features accounted for 9.3%. At the individual-feature level, subtalar angle ROM, Hardness, hip flexion ROM, knee valgus ROM, hip rotation, and RMS ST showed relatively high mean absolute SHAP values. The SHAP summary plot further displayed the distribution of feature contributions across individual samples. In this plot, positive SHAP values indicated contributions toward classification as elevated VALR-related loading, whereas negative SHAP values indicated contributions toward classification as lower-loading samples. These SHAP results were therefore interpreted as exploratory model-based feature contributions, rather than evidence of direct biomechanical causality.

## 4. Discussion

This study focuses on elevated VALR-related loading in school-aged boys during the stance phase of forefoot strike (FFS) running under different midsole hardness conditions. The overall results suggest that, with increasing midsole hardness, VALR showed an overall increasing pattern, but no statistically supported change point was identified. Therefore, the loading response in this study should be interpreted as a continuous trend across hardness conditions rather than as evidence of a fixed elevated VALR-related loading threshold. In addition, the machine-learning and SHAP results suggest that the classification of elevated VALR-related loading was not mainly reflected by an abnormal change in a single indicator but was more likely based on an integrated model-based pattern involving multiple biomechanical features.

Regarding the definition of the loading response, the results show that VALR changes relatively smoothly from 37 Shore C to 42 Shore C, whereas its mean value shows the largest descriptive increase between 42 Shore C and 47 Shore C. However, adjacent pairwise comparisons showed no significant differences after Holm correction, and the linear model without a change point had the lowest AIC. Therefore, 47 Shore C should not be defined as a statistically supported critical hardness threshold. Rather, this result suggests that school-aged boys may show a tendency toward higher impact loading under harder midsole conditions, but the present data only support an overall increasing trend rather than a definite threshold effect [7,19]. One possible explanation is that the abilities of school-aged children to attenuate impact at foot contact and regulate lower-limb elasticity are still developing. However, because no clear change point was identified, this explanation should be interpreted cautiously and regarded as a possible developmental factor rather than direct evidence of a threshold mechanism.

The multi-model analysis shows that, under participant-level GroupKFold validation, XGBoost achieved the highest accuracy, precision, and F1-value among the tested models, whereas KNN showed the highest recall and Random Forest showed the highest pooled out-of-fold AUC. Therefore, XGBoost should not be described as achieving the best performance across all evaluation metrics. Nevertheless, XGBoost was retained for the subsequent SHAP analysis because of its overall classification performance and compatibility with model-based feature interpretation. The advantage of using a tree-based ensemble model remains relevant to the biomechanical complexity of the task itself. When school-aged boys perform FFS running under different midsole hardness conditions, lower-limb regulation of impact loading is unlikely to follow a simple linear change in a single variable. Instead, it may involve nonlinear coupling among neuromuscular activation, multijoint kinematic adjustment, and multi-plane compensation. In this context, XGBoost can rely on tree-based ensembles to capture nonlinear classification boundaries and feature interactions. However, under the stricter participant-level validation framework, the model showed moderate rather than strong discriminative performance. This result indicates that the classification of elevated VALR-related loading remains challenging in a small pediatric sample and should be interpreted cautiously [29,51,53].

From the perspective of feature contribution, the exploratory SHAP analysis further shows that, when school-aged boys perform FFS running under different midsole hardness conditions, the model does not identify elevated VALR-related loading mainly on the basis of proximal sagittal-plane postural changes. Instead, it relies more on information provided by distal and non-sagittal kinematic features and electromyographic features. Specifically, the cumulative contribution of distal and non-sagittal kinematic features reached 65.0%, followed by electromyographic features at 25.7%, whereas proximal sagittal-plane features accounted for 9.3%. This result suggests that, in the present classification task, the model depends more on information related to distal motor regulation, multi-plane joint control, and muscle recruitment than on any single local postural indicator [54,55]. To some extent, this finding also extends earlier approaches that place greater emphasis on single-joint and single-plane indicators in the assessment of running load, but it should be understood as a model-based exploratory result rather than a confirmed biomechanical mechanism.

At the level of individual features, subtalar angle ROM, Hardness, hip flexion ROM, knee valgus ROM, hip rotation, and RMS ST showed relatively high mean absolute SHAP values. However, it should be noted that the high ranking of these features mainly reflects the model’s use of the discriminative information contained in these variables, rather than implying that any one of these indicators alone can define elevated VALR-related loading independently of other variables. In particular, the appearance of Hardness among the highly ranked features indicates that shoe condition still provided useful information to the model, even though no statistically supported hardness threshold was identified. At the same time, except for the highest-ranked features, the contribution differences among other features remained relatively gradual rather than forming a clearly separated hierarchy. This pattern indicates that the model’s discriminative ability was not concentrated in a few decisive variables but depended on the joint contribution of multiple features.

Further evidence from the SHAP scatter beeswarm plot shows that although distal and non-sagittal kinematic features and electromyographic features dominated the overall contribution, their values did not show a consistent directional relationship with the model output. For most high-contribution features, both high-value and low-value samples appeared in the positive and negative SHAP regions, and some features showed substantial overlap around 0. This pattern indicates that the model did not rely on these variables through a simple linear or monotonic relationship but was more likely influenced by between-sample variation and the joint configuration of other input variables. Therefore, even when a feature makes a large overall contribution, it cannot be interpreted as meaning that an increase or decrease in its value necessarily corresponds to a higher probability of elevated VALR-related loading. It should also be noted that SHAP analysis reveals the associative contribution of feature values to model output rather than direct causal relations among variables. Therefore, the interpretation above is better understood as describing exploratory model-based feature contributions under the present task conditions, rather than direct evidence that the related biomechanical factors are involved in the formation mechanism of elevated loading [29,53].

In summary, the present results suggest that increasing midsole hardness was associated with an overall upward trend in VALR during FFS running in school-aged boys, but 47 Shore C should not be interpreted as a statistically supported critical threshold. At the same time, the combination of XGBoost and SHAP can classify elevated VALR-related loading with moderate performance and suggests that the underlying discrimination depends more on coordinated contributions across multiple features than on an abnormality in any single indicator. This finding provides preliminary data support for understanding the multi-feature discriminative characteristics of elevated VALR-related loading during the stance phase of children’s FFS running.

However, this study still has several limitations. First, the elevated-loading label is defined mainly on the basis of the median VALR value across valid trials. Although VALR has clear implications for impact loading, it cannot fully capture the multidimensional nature of running-related load risk, and the present label should not be interpreted as a clinical injury-risk threshold. Future studies may incorporate additional kinetic, kinematic, electromyographic, and prospective injury-related indicators to build a more comprehensive risk evaluation framework [56]. Second, this study is conducted under specific sample conditions and a laboratory testing setting, and the external applicability of the results still requires further validation in larger samples, across different age stages, and in tasks that more closely approximate real sports environments [57]. In particular, the relatively small sample size of 14 participants limits the statistical stability of the findings and may restrict the generalizability of the results to broader pediatric populations. Moreover, only school-aged boys within a limited age range were included, and therefore, the present findings cannot be directly extended to girls, children at other developmental stages, or adolescent runners. Another limitation is that biological maturity was not assessed in the present study. Although participants were within a limited chronological age range, children of similar chronological age may differ in maturation status, which may influence lower-limb biomechanics and neuromuscular control. Future studies should include biological maturity indicators, such as maturity offset, peak height velocity estimation, or skeletal age, to better account for maturation-related variability. Third, although participant-level GroupKFold validation was used to reduce information overlap among trials from the same participant, the number of participants remained limited, and model robustness still needs to be evaluated in independent pediatric cohorts. In addition, the use of a single force platform limited the analysis to valid dominant leg stance trials and may restrict the assessment of bilateral or step-to-step loading patterns. Fourth, although multiple valid running trials were collected under each hardness condition, the study was based on a single laboratory testing session. This design does not allow assessment of day-to-day variability, adaptation over repeated exposure, or the long-term effects of different midsole hardness conditions on running biomechanics and impact loading. Future studies should include repeated measurements and longitudinal follow-up designs to determine whether the identified biomechanical patterns remain stable over time. The controlled running speed should also be considered when interpreting the findings. Running speed was maintained within a relatively narrow jogging range of approximately 2.0–2.3 m/s rather than allowing each child to run freely at a fully self-selected speed. Because VALR is speed-dependent, the absolute VALR values and the corresponding elevated-loading labels may be influenced by the controlled speed range. Therefore, the present findings should be interpreted within this standardized low-to-moderate running-speed condition, and future studies should examine whether similar biomechanical patterns are observed under self-selected and faster running speeds. Finally, the SHAP results reflect the contribution of features to model output rather than biomechanical causal relations [58]. In addition, potential multicollinearity among the 28 biomechanical input features was not systematically modeled in the present analysis, which may influence the stability and independence of individual SHAP-based feature interpretations. Therefore, the SHAP results should be interpreted as exploratory model-based feature contributions rather than independent causal effects of single variables. Future work still needs longitudinal studies or intervention experiments to further examine the actual relationship between these key features and elevated VALR-related loading.

## 5. Conclusions

This study examines elevated VALR-related loading during the stance phase of FFS running in school-aged boys under different midsole hardness conditions from three aspects, namely VALR response analysis, participant-level classification model identification, and exploratory feature contribution interpretation. The results show that as midsole hardness increases, VALR during the stance phase of FFS running in school-aged boys generally increases, but no statistically supported change point is identified. Therefore, elevated-loading labels were defined using trial-level VALR values rather than a fixed midsole hardness threshold. The model comparison results show that XGBoost demonstrates moderate discriminative performance under participant-level validation and is retained for exploratory SHAP analysis because of its overall classification metrics and interpretability. Further SHAP analysis shows that the classification of elevated VALR-related loading does not mainly depend on an abnormal change in a single indicator but is more likely related to the coordinated contribution of multiple biomechanical features. Among these features, distal and non-sagittal kinematic features and electromyographic features contribute more, whereas proximal sagittal-plane features contribute relatively less. This study preliminarily reveals the multi-feature discriminative pattern of elevated VALR-related loading during the stance phase of FFS running in school-aged boys under different midsole hardness conditions. It may provide preliminary data support for evaluating running shoe midsole hardness and multidimensional biomechanical responses in school-aged boys, potentially offering a reference for subsequent studies on related mechanisms.

## Figures and Tables

**Table 1:** VALR results under different midsole hardness conditions.

Midsole Hardness (Shore C)	Valid Trials (n)	VALR ($BW\cdot s^{-1}$, Mean ± SD)
37	81	19.07 ± 4.56
42	84	20.13 ± 5.31
47	89	23.83 ± 10.50
52	82	24.02 ± 10.16

VALR denotes vertical average loading rate; BW denotes body weight; SD denotes standard deviation. Valid Trials (*n*) refers to the number of valid trials included under each midsole hardness condition, rather than the number of participants.

**Table 2:** Adjacent pairwise comparisons and candidate change-point model comparison for VALR across midsole hardness conditions.

Analysis	Result
37 vs. 42 Shore C	pHolm = 1.000, not significant
42 vs. 47 Shore C	pHolm = 1.000, not significant
47 vs. 52 Shore C	pHolm = 1.000, not significant
Linear model without change point	AIC = 885.22, ΔAIC = 0.00
Piecewise model, knot at 42 Shore C	AIC = 887.21, ΔAIC = 2.00
Piecewise model, knot at 47 Shore C	AIC = 886.24, ΔAIC = 1.02

VALR, vertical average loading rate; AIC, Akaike information criterion. Pairwise *p* values were adjusted using the Holm correction. ΔAIC indicates the difference from the best-fitting model; lower AIC values indicate better model fit.

**Table 3:** Participant-level performance comparison of different classification models for elevated VALR-related loading classification.

Models	Acc	Pre	Rec	F1-Value	AUC
XGBoost	75.93%	74.14%	79.63%	0.768	0.738
RF	71.30%	72.55%	68.52%	0.705	0.746
MLP	42.13%	41.79%	51.85%	0.462	0.421
KNN	64.81%	60.00%	88.89%	0.716	0.659
LR	47.22%	47.17%	46.30%	0.467	0.502
SVM	52.78%	53.85%	38.89%	0.452	0.527

Acc denotes accuracy; Pre denotes precision; Rec denotes recall; AUC denotes area under the curve.

## Data Availability

The raw data supporting the conclusions of this article will be made available by the authors on request.

## Abbreviations

The following abbreviations are used in this manuscript:

VALR	Vertical Average Loading Rate
FFS	Forefoot Strike
SHAP	Shapley Additive Explanations
sEMG	Surface Electromyography
XGBoost	Extreme Gradient Boosting
RMS	Root Mean Square

## Appendix A

**Table A1:** List of the 28 biomechanical input features used for machine-learning classification.

Feature Group	Feature Name
Distal and non-sagittal kinematics	Knee valgus angle
Distal and non-sagittal kinematics	Knee valgus range of motion
Distal and non-sagittal kinematics	Metatarsophalangeal angle
Distal and non-sagittal kinematics	Metatarsophalangeal range of motion
Distal and non-sagittal kinematics	Hip rotation angle
Distal and non-sagittal kinematics	Hip rotation range of motion
Distal and non-sagittal kinematics	Hip adduction angle
Distal and non-sagittal kinematics	Hip adduction range of motion
Distal and non-sagittal kinematics	Subtalar angle
Distal and non-sagittal kinematics	Subtalar range of motion
Distal and non-sagittal kinematics	Ankle angle
Distal and non-sagittal kinematics	Ankle range of motion
Distal and non-sagittal kinematics	Knee angle
Distal and non-sagittal kinematics	Knee range of motion
EMG features	RMS of semitendinosus
EMG features	RMS of medial gastrocnemius
EMG features	RMS of gluteus maximus
EMG features	RMS of erector spinae
EMG features	RMS of tibialis anterior
EMG features	RMS of biceps femoris
EMG features	RMS of vastus lateralis
EMG features	RMS of vastus medialis
EMG features	RMS of rectus femoris
EMG features	RMS of lateral gastrocnemius
Proximal sagittal kinematics	Hip flexion angle
Proximal sagittal kinematics	Hip flexion range of motion
Proximal sagittal kinematics	Knee flexion angle
Proximal sagittal kinematics	Knee flexion range of motion
